# Research Progress and Hot Spot Analysis of the Propagation and Evolution Law of Prefabricated Cracks in Defective Rocks

**DOI:** 10.3390/ma16134623

**Published:** 2023-06-27

**Authors:** Shu Zhu, Zhende Zhu, Luxiang Wang, Junyu Wu

**Affiliations:** 1 Key Laboratory of Ministry of Education of Geomechanics and Embankment Engineering, Hohai University, Nanjing 210098, China; 20210940@hhu.edu.cn (S.Z.); wjy1995@hhu.edu.cn (J.W.); 2 Jiangsu Research Center for Geotechnical Engineering, Hohai University, Nanjing 210098, China

**Keywords:** crack propagation, knowledge structure, bibliometrics, CiteSpace

## Abstract

The generation of rock mass disasters in underground engineering essentially arises from the disruption of the original three-dimensional stress equilibrium of the rock mass caused by excavation and other activities, leading to the redistribution of stress fields. During the excavation process, the engineering rock mass undergoes complex dynamic stress equilibrium processes involving loading and unloading. This equilibrium process promotes the nucleation, initiation, and propagation of pre-existing cracks in the surrounding rock, resulting in changes in the internal structure of the rock mass and a weakening of its strength. Eventually, this localized cracking extends to global failure. In order to understand the current status better and study the development trends in the study of crack propagation and evolution in defective rock, this study conducts a bibliometric analysis of 288 articles from the Web of Science Core Collection database using CiteSpace software (version 6.1.R4). The results indicate an increasing trend in the annual publication output, characterized by two phases of emergence and rapid development. The countries of China, the United States, and Iran have the highest publication output in this field. The most frequently cited journals include INT J ROCK MECH MIN, ENG FRACT MECH, and ROCK MECH ROCK ENG. This study provides a comprehensive analysis of the current status and development trends in the research on the propagation and evolution of pre-existing cracks. This study enhances the comprehension of crucial aspects of crack propagation and evolution in rock materials with defects. Moreover, it opens up new possibilities for future investigations and holds promising implications for researchers and practitioners in the field.

## 1. Introduction

The process of rock failure is the cumulative damage and fracture process resulting from the initiation, propagation, and coalescence of internal cracks within the rock [1,2,3,4]. Understanding the mechanisms of crack propagation and coalescence in rocks is of significant importance for studying rock failure [5]. In rock mechanics, the geometry, orientation, and distribution of internal fractures in fractured rock masses typically govern the deformation and strength of the rock mass [6]. The instability and failure of fractured rock masses are closely related to the opening, propagation, and coalescence of these fractures [7,8,9]. In geophysics, laboratory experiments involving rocks and other materials are an important way to study the formation and evolution processes of faults. Explaining the mechanisms and interactions involved in fault formation from a mechanical perspective has always been a challenging theoretical problem in structural geology [10,11,12].

It is well known that the apparent failure phenomena of engineering rock masses [13], such as landslides, collapses, rockfalls, tilting, bottom heaving, and roof caving, can be described using different strength theories in rock mechanics [14,15,16]. The commonly used rock strength theories include the maximum normal stress theory, the maximum shear stress theory, the octahedral shear stress theory, the Mohr theory, the Griffith theory, and the Lode–Bishop theory. In addition, the rock failure criteria encompass the Mohr–Coulomb criterion, the D–P criterion, empirical failure criteria, and empirical criteria for the propagation of compression-shear cracks [17,18]. However, these theories and criteria have their own applicability conditions and cannot fully explain the deformation and failure modes of macroscopic rocks. With the further development of rock mechanics and engineering, to establish constitutive relationships and failure criteria that truly reflect the behavior of rocks, it is necessary to understand the mechanisms of crack initiation, nucleation, propagation, coalescence, and evolution leading to failure within the rocks. This is because rock failure occurs when localized microcracks propagate to macroscopic cracks, develop into coalescence, and eventually reach overall instability [19]. Solving this problem requires experimental research that reproduces the deformation and strength degradation patterns of rocks in complex environments, as well as the process of crack propagation and coalescence. Therefore, the study of crack initiation, propagation, coalescence, fracture processes, fracture patterns, and the evolution of crack spatial distribution in engineering rock masses containing initial defects has been a hot and challenging topic in the field of rock mechanics and engineering. Its resolution undoubtedly promotes the development of rock damage mechanics theory.

Over the past years, using bibliometrics as a quantitative analytical approach has gained extensive popularity in unveiling publication distribution, disciplinary attributes, and significant contributors from both institutions and authors, as well as identifying research trends and frontiers within specific research domains. [20]. Based on visual analysis using CiteSpace, Deng et al. [21] investigated rock mechanics related to freeze–thaw phenomena from 2013 to 2020. It was concluded that the coupling effects of multiple fields in rock mechanics and the engineering application of mature research outcomes would be the key focus of future freeze–thaw rock mechanics research. Zhao [22] conducted analysis and organizational research on the acoustic emission of steeply inclined coal using bibliometric software. The research findings have mainly been applied in geological surveys, mineral resource extraction, nuclear waste storage, and other related areas. However, the aforementioned studies did not specifically address the crack propagation in defective rock masses, and there are limited explorations regarding the temporal evolution of research hotspots and frontiers in this area.

To bridge the research gap outlined above, the primary objective of this study is to conduct a comprehensive investigation into the current state and emerging trends in pre-existing crack propagation research, employing an integrated bibliometric analysis approach. The subsequent sections of this paper are organized as follows: Section 2 outlines the literature search strategy and the bibliometric methods applied in this study. Section 3 presents the findings of the bibliometric analysis, encompassing publication volume and trends, collaborative networks, and research hotspots. Section 4 critically discusses the current status and prospects of pre-existing crack propagation research. Finally, Section 5 concludes the study by summarizing the key insights and implications derived from the analysis.

## 2. Data and Methods

### 2.1. Data Acquiring and Cleaning

For the literature analysis, this study opted for the Web of Science (WoS) Core Collection as the chosen database. The WoS Core Collection stands as a comprehensive and authoritative literature search engine, encompassing the Science Citation Index Expanded (SCIE), the Social Sciences Citation Index (SSCI), and the Arts & Humanities Citation Index (A&HCI). Renowned for its extensive coverage and reliability, it has been widely employed in diverse fields for literature reviews and bibliometric analysis. By utilizing the WoS Core Collection, this study aims to ensure access to a diverse range of scholarly publications and enable a comprehensive analysis of the research on the evolution and trends in the study of pre-existing crack propagation.

This study retrieved literature using the “All fields” and “Topic” search types in the chosen database. The search included the titles, abstracts, author keywords, and keyword search of the article titles. The search terms used were “crack growth in rocks”, “crack propagation”, and “crack coalescence”. The literature type was limited to “article”. The search period was set from 1996 to 2022, resulting in a total of 295 articles. After excluding irrelevant literature, a final set of 288 relevant articles was obtained. The selected articles were downloaded in the format of “abstracts” and “full records” (including cited references) and saved as plain text files for further analysis.

### 2.2. Analysis Method

The selection of literature metrics software was guided by three primary criteria. Firstly, the software needed to be capable of automatically extracting information from the literature. Secondly, it should offer a diverse range of functions to facilitate a comprehensive analysis of the collected literature from multiple perspectives. Lastly, the software should feature robust visualization capabilities to present the results effectively.

In line with these requirements, CiteSpace (version 6.1.R4) was chosen as the designated tool for conducting literature metrics analysis in this study. CiteSpace, a widely utilized citation visualization analysis software, was developed by Prof. Chaomei Chen [21] and his team at Drexel University. This multifunctional software harnesses techniques such as data mining, information processing, and knowledge measurement to analyze and visualize specific research domains. By employing visualization techniques, it generates insightful knowledge maps, referred to as “knowledge landscapes”, which illuminate the development process and knowledge structure within the field of research.

In this study, the CiteSpace software was employed to conduct the analysis and visualization of the present state and developmental trends concerning crack propagation and coalescence laws in defective rocks. The analysis focused on various aspects, including research disciplines, collaborative networks, and research hotspots. Within the CiteSpace software, different node types were selected to achieve diverse perspectives.

To identify the research disciplines involved in studying crack propagation and coalescence laws in defective rocks, the “Class” node type was utilized. By setting the node types as “Country”, “Institution”, and “Author”, collaborative networks at corresponding levels within the field were explored, shedding light on the collaborative dynamics among different entities. Additionally, noun phrases extracted from titles, abstracts, author keywords (DE), and keyword plus (ID) of the literature were selected as node types for “keyword clustering”. This approach allowed for an examination of the research hotspots during different stages of the entire research period, capturing the evolving focus of the field over time.

## 3. Results

### 3.1. Analysis of Annual Publication Volume

**Figure 1:**
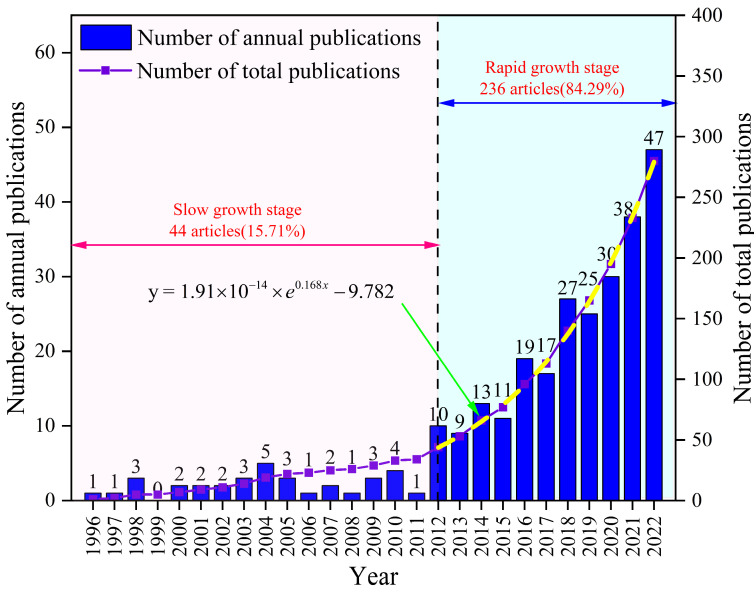
Statistics on the number of publications (1996–2022).

The number of publications can indicate the level of attention in a research field and, to some extent, reflects the overall trend, development speed, and research interest in that field. Based on the retrieved literature, the annual publication trends in the study of pre-existing crack propagation and coalescence laws from 1996 to 2022 were analyzed (Figure 1).

From Figure 1, it can be observed that the number of publications on this topic shows an overall increasing trend each year. Based on the growth rate of annual publications, it can be divided into two phases; the first is a period of slow growth from 1996 to 2011. During this period, the annual publication count remained relatively stable, with an average of two to three publications per year in this research field. From 2012 to 2022, there was a phase of rapid growth. Notably, the number of articles published in 2012 was nearly ten times that of 2011, and the publication count showed a sharp upward trend, reaching its peak in 2022 (47 publications). It can be inferred that the study of pre-existing crack propagation and coalescence laws has received significant attention from the academic community in the past decade and has entered a phase of vigorous development.

### 3.2. Analysis of Cooperation Network

#### 3.2.1. Cooperation Network of Countries

**Figure 2:**
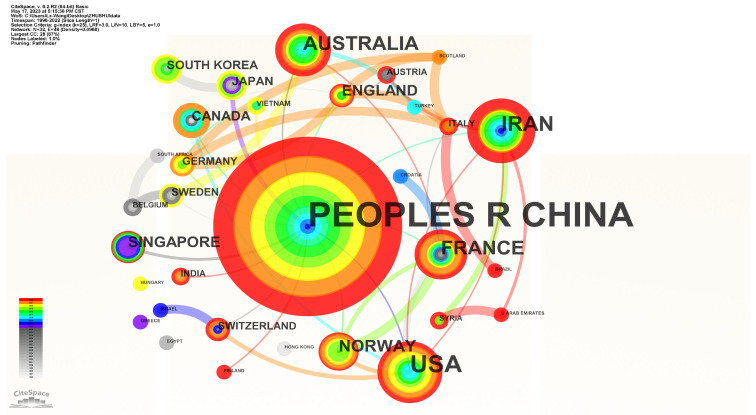
Cooperation Network of Countries.

The collaborative network of countries involved in the study of pre-existing crack propagation and coalescence laws is shown in Figure 2. The size of each node represents the number of publications from a particular country, and the links between nodes indicate the collaboration between these countries. The main contributing countries include China, France, Australia, Iran, the United Kingdom, and Norway. Table 1 lists the top 10 countries that make the largest contributions to the research on pre-existing crack propagation laws and their respective years of initial publication.

China published the highest number of papers, with a total of 196, accounting for 68.05% of the total publications. The United States and Iran follow, with 40 and 22 papers published, respectively. Table 1 also s each countr’s centrality, which measures the number of times a node acts as a bridge between other nodes in the network and is considered an indicator of node importance. The order in Table 1 was based on centrality, from high to low. It can be observed that China rank highest in terms of publication quantity and centrality, indicating that Chinese scholars closely focused on this research field, conducted extensive studies, and exhibited high levels of collaboration with other countries.

#### 3.2.2. Cooperation Network of Institutions

**Figure 3:**
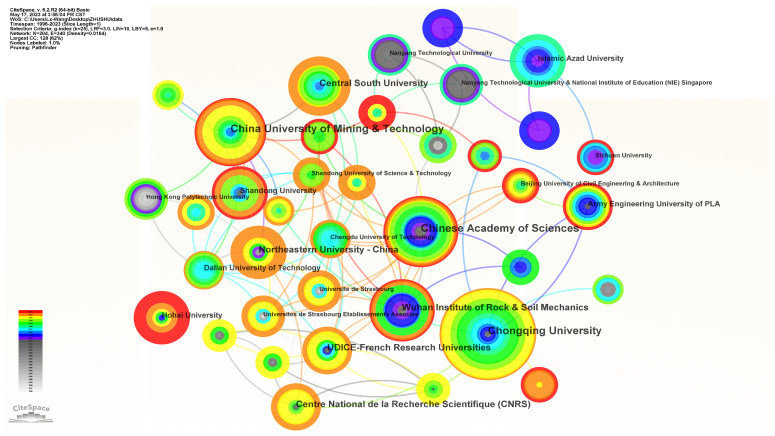
Cooperation network of institutions.

The distribution of publications among different research institutions is shown in Figure 3. Each node represents an institution, and the node size corresponds to the number of publications. Table 2 lists the top 10 contributing research institutions and the year of their first appearance. Among them, the institution with the highest publication output is Chongqing University, with a total of 31 papers, accounting for 10.76% of the total publications in the Web of Science search results. China Academy of Sciences (24 papers) and China University of Mining and Technology (22 papers) follow in publication quantity. Regarding centrality, China University of Mining and Technology rank first, indicating its high level of activity in the research field of pre-existing crack propagation and coalescence laws.

#### 3.2.3. Cooperation Network of Authors

**Figure 4:**
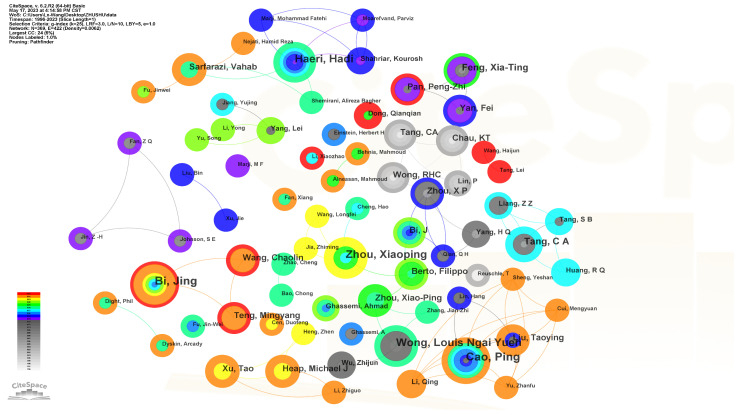
Cooperation network of authors.

The author collaboration network was constructed, as shown in Figure 4. Each node represents an author, and the size of the node represents the author’s contribution. Specifically, Zhou Xiaoping is the top author with the highest number of publications as the first author (15 papers). Similarly, Cao Ping, Wong Louis Ngai Yuen, Haeri Hadi, Tang Chunan, Feng Xiating, Li Shucai, and others are also experts in this field.

### 3.3. Analysis and Discussion of Co-Citation

#### 3.3.1. Journals of Co-Cited

**Figure 5:**
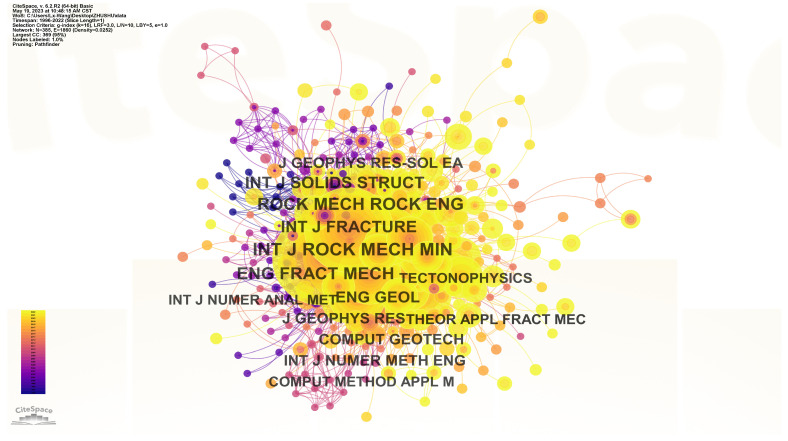
Network of co-cited journals.

The analysis of journal co-citations can reflect the academic contributions and influence of research on the expansion laws of prefabricated cracks from the perspective of different journals. The co-cited journal network was generated, as shown in Figure 5. Each node represents a major co-cited journal, and the size of the node reflects the co-citation frequency of that journal. Table 3 displays the co-citation frequency, impact factor, and rankings of the top 10 journals based on the publishers. The journal “INT J ROCK MECH MIN” is the most frequently cited journal in the research on the expansion of prefabricated cracks, with a co-citation frequency of 262. Similarly, this journal is also one of the top journals in the geotechnical engineering field, consistently ranking high in terms of impact factor and industry influence. Other influential journals include “ENG FRACT MECH” (218 citations) and “ROCK MECH ROCK ENG” (200 citations). These journals made significant contributions to the study of the expansion laws of prefabricated cracks.

#### 3.3.2. Co-Cited Literature

**Figure 6:**
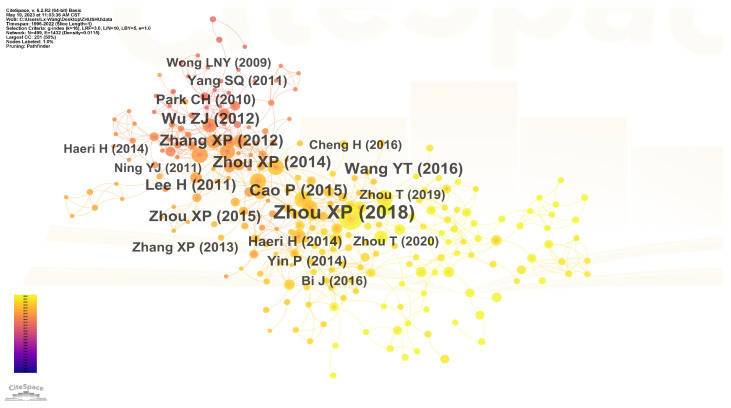
Network of co-cited references.

Analyzing co-citations makes it possible to identify influential papers and provide a comprehensive overview of the major academic accomplishments within the research field [23]. In Figure 6, the co-cited article network in the domain of prefabricated crack expansion laws is presented. Each node in the network represents a research paper, with the size of the node indicating the frequency of citations received by that particular paper. This visualization offers valuable insights into the significance and impact of individual research papers within the field. Table 4 lists the top 5 highly cited references.

From the perspective of research hotspots, most of the top five highly cited articles are focused on the study of the expansion mechanisms of prefabricated cracks. Zhou et al. [24] investigated the crack initiation, propagation, coalescence, and trapping of two intersecting three-dimensional embedded flaws in PMMA specimens under uniaxial compression conditions. They explored the major differences between the behaviors of 2D and 3D cracks in pre-existing flaws, emphasizing these aspects for the first time. Cao et al. [25] studied fracture coalescence by loading rock-like specimens with two and three pre-existing flaws. The research revealed that with an increasing angle of the rock bridge, the failure mode transitions from crack propagation failure to crack coalescence failure, and this transition becomes more prominent with larger angles of prefabricated cracks. Building upon previous studies on single and double cracks, Zhou et al. [26] conducted uniaxial compression experiments on rock-like materials with multiple flaws to investigate the influence of pre-existing flaw layouts on mechanical properties further, crack initiation patterns, and types of crack coalescence. Wu et al. [27] formulated the physical problem using both physical and mathematical grids and employed the numerical manifold method’s contact technique and the MohrCoulomb crack initiation criterion to study the influence of friction and cohesion on the propagation of closed flaws (cracks) under compressive conditions. Wang et al. [28] simulated the initiation, propagation, and merging of pre-existing flaws in rocks under compressive loading using the extended non-ordinary state-based peridynamics (NOSB-PD) approach.

### 3.4. Keywords Analysis

**Figure 7:**
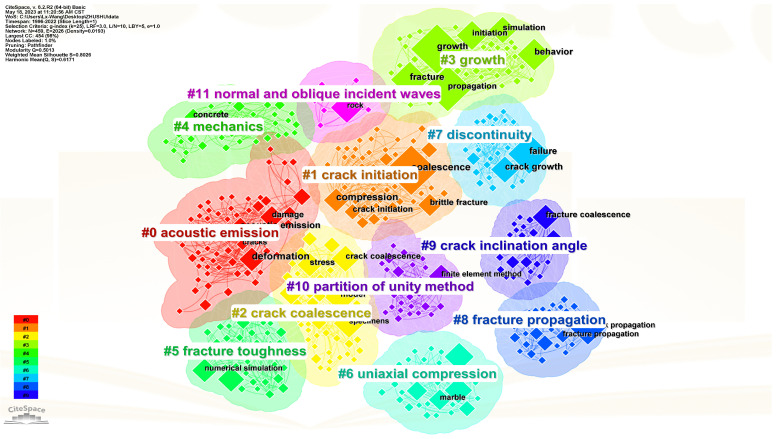
Keyword clustering network.

**Figure 8:**
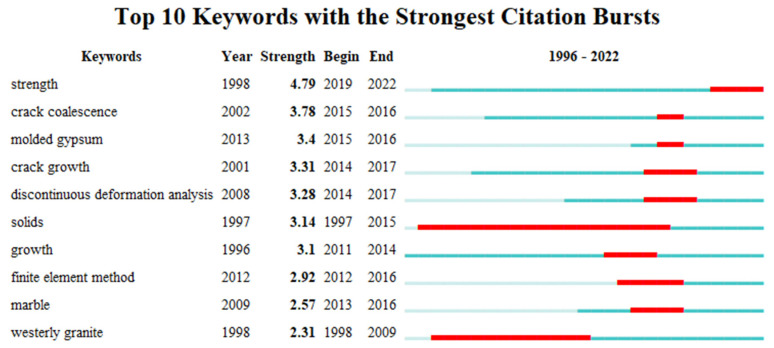
Keyword clustering sorted by intensity.

**Figure 9:**
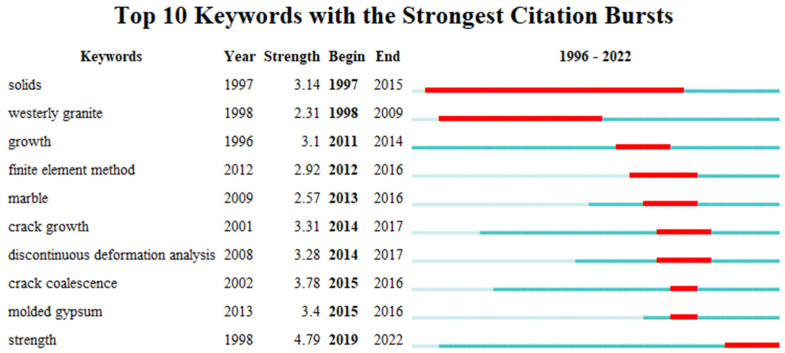
Keyword clustering sorted by time.

The keywords of the literature play a crucial role in summarizing the core content of an article and analyzing the trends and hotspots in scientific research. Analyzing the keywords of the literature helps researchers grasp the dynamics of scientific research quickly. Using CiteSpace software, a total of 252 terms with a frequency of five or more occurrences were detected. Figure 7 visualizes the keyword clustering network throughout the research period, where each node represents a keyword, and the size of the node reflects the frequency of occurrence of that term. Additionally, the links between different nodes indicate the strength of co-occurrence between two keywords. The research hotspots in the study of prefabricated crack expansion mechanisms mainly focused on the following aspects: expansion processes, failure modes, and mechanical behaviors. Figure 8 summarizes the top 10% of keywords in terms of their appearance in each year and the year of their first appearance, aiming to explore more detailed information on research hotspots and reveal the temporal evolution of research topics. From Figure 9 it can be observed more intuitively that the frequency of new keyword appearances was higher from 1996 to 2000 and gradually decreased thereafter. Furthermore, since 2014, despite the exponential growth in the number of publications, the emergence of new keywords has significantly declined. This phenomenon can be attributed to the comprehensive identification of relevant keywords in the initial stages of the research. By analyzing high-frequency keywords, research priorities can be effectively captured from various angles, including research content, research methods, research subjects, and research contexts. This multi-perspective approach provides valuable insights into the key areas and aspects of the research, enabling a more holistic understanding of the field.

**Figure 10:**
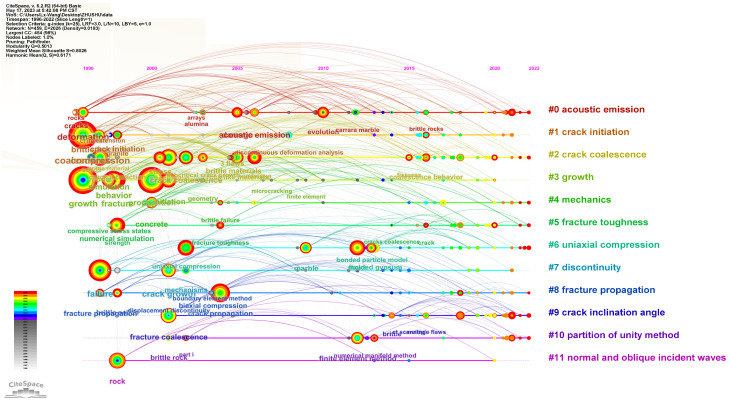
Keyword clustering time network.

Figure 10 displays the timeline of the top 10 most significant clusters in the study of prefabricated crack expansion and evolution patterns. Among them, “acoustic emission” has the longest duration, spanning the entire research period from 1996 to the present. This article considers acoustic emission technology as an important technique for studying the evolution patterns of prefabricated crack expansion. “Crack initiation” and “crack coalescence” are crucial factors influencing prefabricated crack expansion, and these two keywords are extensively discussed later in the article.

## 4. Discussion

### 4.1. Current Research Focuses

This study reveals the focus and development trajectory of research on the evolution patterns of prefabricated crack expansion through the analysis of keyword co-occurrence networks and keyword clustering evolution in this field. In Section 3.4 of this paper, the research status is discussed in three aspects: the preparation materials of prefabricated cracks, the main technical approaches, and the mechanisms of prefabricated crack expansion.

Due to the heterogeneous and brittle nature of rocks, their failure process is relatively short compared to metallic materials. The limited understanding, lack of research methods, and short duration of failure have led people to overlook the development and expansion of inherent defects within rocks, focusing instead on the macroscopic aspects of crack propagation, coalescence, and ultimate failure. As the understanding gradually deepened, the knowledge of the early stages of rock failure transitioned from a macroscopic scale to a microscopic scale. Researchers began to summarize the expansion patterns from a microscopic perspective. From Figure 9, it can be observed that the period between 1998 and 2013 focused on studying intact rocks such as granite and marble. However, the preparation of cracks within samples was extremely challenging. Therefore, for convenience, scholars began using rock-like materials instead of natural rocks in their research, starting around 2015. Materials such as PMMA, gypsum, ceramics, and resins were widely employed. These rock-like materials not only needed to exhibit similar brittleness and compression-to-tension ratios as natural rocks but also had to possess similar physical and mechanical properties. The main emphasis was on the shear characteristics under uniaxial compression conditions. Wong T.F. [29,30] conducted compression tests on Westerly granite specimens with pre-existing microcracks, observing the phenomenon of reverse wing cracks and analyzing the mechanism behind them. Reyes et al. [31] conducted uniaxial compression tests on gypsum samples with two pre-existing cracks, providing insights into the path of complete specimen failure. Ashby [32] and Nemat-Nasser [33] performed physical model compression tests using similar materials with single and multiple cracks, observing the initiation and propagation shapes of surface-penetrating cracks and rock bridge penetration patterns, revealing the evolution of crack propagation. Zhu et al. [34] conducted biaxial experiments under water pressure using transparent resin-based rock-like materials, revealing the evolution patterns of prefabricated crack expansion.

In the CiteSpace analysis, “acoustic emission” was observed as the largest keyword cluster. Introducing this technique in rock mechanics experiments undoubtedly provided a new approach to exploring the research on prefabricated crack propagation in rocks. This point was confirmed through the analysis of highly cited literature earlier. Acoustic emission technology was introduced to the discipline of geotechnical engineering in 1996 [35,36,37]. Due to the inadequate understanding of complex rock structures, especially how the internal structure of rocks changes during stress state alterations, it became a topic of concern for rock mechanics researchers. Combining acoustic emission technology with rock rigid servo systems added a new perspective to understanding rock structure failure. Currently, many scholars use acoustic emission technology to conduct experimental research on fracture instability in rocks. Scholz [38] studied the acoustic emission patterns in the pre-failure rock expansion phenomenon. Goodman [39] investigated the Kaiser effect of acoustic emission by conducting fully confined compression loading tests on sandstone and quartzite and analyzing the results in conjunction with the acoustic emission ring count. Liu Dongyan et al. simulated rocks using cement mortar-based similar materials and performed uniaxial compression tests on specimens. They studied the entire process of initial crack growth, expansion, and breakthrough to failure by studying the acoustic emission ring counts. The different stages of crack initiation, propagation, and breakthrough were distinguished by inflection points on the acoustic emission process curve. The results indicated that the majority of acoustic emission ring counts during the compressed failure process came from the energy released during the growth of newly formed cracks at the crack tip. Chang S.H. et al. [40] conducted pseudo-triaxial compression tests on marble and granite specimens using acoustic emission technology. They analyzed rock stress–strain indicators and corresponding curves of acoustic emission ring counts to address the timing of microcrack initiation and coalescence, as well as the predominance of shear failure in rocks with increasing confining pressure. Zhao et al. [41] conducted loading–unloading tests on rock specimens of different sizes using acoustic emission technology and discussed the influence of size and loading–unloading methods on fracture patterns. Lei et al. [42] performed compression–unloading tests on mudstone and granite specimens with pre-existing fractures, recorded acoustic emission ring counts during the progressive failure process, and obtained the relationship between crack initiation, propagation, and the number of ring counts. Li Shucai et al. [43] conducted triaxial compression tests with different loading–unloading stress–strain processes on 50 sandstone specimens using both acoustic emission and resistivity methods. They quantitatively analyzed the experimental data and used a damage variable that included resistivity and ring counts to describe the evolution of rock damage.

The keywords “crack initiation” and “crack coalescence” indicate that current research typically starts from the perspective of classical mechanics to explore the initiation and coalescence of internal defects in metal and rock materials, which leads to the degradation of material strength within the framework of discontinuous solid mechanics. This also includes keywords such as “strength” and “discontinuity”. In the late 1980s, Kyoya [44,45] introduced the concept of damage to analyze the damaged zone in surrounding rock mass calculations for underground excavations. Kawamoto [46] incorporated anisotropic fracture damage theory into the mechanical study of discontinuous rock mass using a second-order symmetric tensor. The establishment of constitutive theory for fractured rock masses inevitably involves the manifestation of internal defect variations in the constitutive relationship, which relates to the study of “fracture toughness” (keyword 5) in rocks. Zhou et al. [10] calculated the stress intensity factor of multiple fissures under compression-shear stress. Erdogan and Sih [47] proposed a fracture initiation criterion under mixed-mode loading, which starts from a stress perspective. According to this criterion, when the tangential stress reaches a specific value, the crack will initiate along the direction of maximum tangential stress, leading to material instability and propagation. Zhou Xiaoping et al. [3], using the Dugdale–Barenblatt constitutive model, conducted numerical simulations to analyze the relationship between crack propagation, breakthrough failure modes, and factors such as stress state, crack spacing, and size.

From this, it can be seen that the initiation, propagation, and coalescence of pre-existing cracks are intrinsic factors causing the failure of engineering rock masses. Although a considerable amount of productive research has been conducted by scholars both domestically and internationally in this field, there is still much work to be done.

### 4.2. Future Prospects

Against the backdrop of ongoing advancements in deep rock engineering, the study of the propagation laws of pre-existing cracks is expected to continue expanding in the future. The following outlook is proposed for future research, aiming to inspire researchers and practitioners and provide new insights to promote the development and progress of this field.

In terms of the study on crack propagation mechanisms, most researchers have focused on two-dimensional analyses without distinguishing between Mode I and Mode II fractures during rock compression. While many researchers have provided relatively accurate results for the initiation angle of cracks (keyword 9), different stages of crack propagation in rock exhibit distinct crack morphologies. However, there is limited research on obtaining the entire three-dimensional dynamic process of secondary crack propagation and accurately determining the crack propagation path during rock expansion. Therefore, studying the complete process of pre-existing crack propagation is significant for rock engineering construction and disaster prevention.

Furthermore, the current research on the expansion mechanism of pre-existing cracks is limited by the use of a single approach. It is worth paying further attention to the in-depth integration of techniques such as acoustic emission and CT scanning with mechanical loading devices like rock servo machines. AE technology enables accurate and comprehensive non-destructive imaging of the internal structural characteristics of rocks, thereby revealing the complex morphology of pre-existing crack propagation and uncovering the internal evolution of rock expansion. However, most CT scanning is performed on unloaded specimens after the completion of experiments, making it impossible to obtain real-time CT images of the entire process of specimen failure. By effectively combining CT scanning with the loading system, it becomes possible to observe the real-time evolution of pre-existing crack propagation and failure throughout the entire process. Therefore, the integration of techniques such as AE or CT scanning with mechanical loading devices like rock servo machines holds great potential for further research in the future.

## 5. Conclusions

This study conducted a comprehensive review of the research status and development trends in the field of pre-existing crack propagation by retrieving 288 papers published between 1996 and 2022 from the WoS Core Collection database. CiteSpace software was used for bibliometric analysis to depict the research landscape from various perspectives, including publication quantity and trends, disciplinary distribution, countries, collaborative networks among institutions and authors, co-citation analysis of journals and references, and term analysis.

The research indicates that the development of pre-existing crack propagation studies shows an upward trend, with two distinct phases, an initial stage and a rapid growth stage, as evidenced by the publication volume. Furthermore, among all countries involved in this field, China, the United States, Iran, and Australia have contributed the most publications. Chongqing University, the Chinese Academy of Sciences, China University of Mining and Technology, Wuhan Institute of Rock and Soil Mechanics, and Central South University have shown the highest number of paper publications. In terms of co-citation analysis, top-cited journals include INT J ROCK MECH MIN, ENG FRACT MECH, ROCK MECH ROCK ENG, INT J FRACTURE, and INT J SOLIDS STRUCT. The most influential paper is the one published in 2018 by Zhou et al., which focuses on the crack initiation, propagation, coalescence, and interaction of two three-dimensional embedded defects in PMMA specimens under uniaxial compression conditions, emphasizing the significant differences between 2D and 3D crack behavior of pre-existing defects.

Based on keyword clustering analysis, acoustic emission technology emerged as an important approach for studying pre-existing crack propagation. Research on crack initiation and coalescence has become a long-standing topic influencing the understanding of crack propagation laws. Further investigation into the dynamic expansion of three-dimensional cracks and the development of research techniques are identified as future research directions in this field.

This study provides a comprehensive overview of the research status and development process of pre-existing crack propagation, filling the gaps in previous research. It helps researchers grasp the mainstream research topics in the current field and provides insights into future research prospects.

While this study has made contributions, there are certain limitations that should be acknowledged. The literature collection in this study was primarily derived from the WoS Core Collection database, which focuses on English-language publications. Consequently, publications from other databases and non-English sources were not included in the analysis. Additionally, non-academic documents like book reviews, government policies, and publications from other authoritative institutions were also excluded. In order to achieve a more comprehensive analysis, it is essential to consider multiple databases that encompass a diverse range of document types and languages. By incorporating a broader selection of sources, a more inclusive and comprehensive understanding of the research topic can be attained. Additionally, the results from the WoS Core Collection are heavily influenced by the settings applied in the search strategy. As research in the field continues to evolve, the search strategy can involve more keywords and document types to gather more detailed information. Moreover, there are several alternative bibliometric analysis tools available, such as VOSviewer, HistCite, and SATI, among others. Exploring a combination of different tools can enhance the performance of literature analysis and visualization.

## Figures and Tables

**Table 1:** Top 10 major contributing countries by number of publications.

NO.	Country	Centrality	Publication	Year
1	PEOPLES R CHINA	0.94	196	1998
2	USA	0.08	40	1996
3	IRAN	0.25	22	2014
4	AUSTRALIA	0.13	18	2012
5	FRANCE	0.18	17	1997
6	NORWAY	0.00	10	2012
7	ENGLAND	0.22	8	1998
8	SINGAPORE	0.00	8	2012
9	CANADA	0.00	6	1998
10	JAPAN	0.11	5	2002

**Table 2:** Top 10 major contributing institutions by number of publications.

NO.	Institution	Publication	Centrality	Year
1	Chongqing University	31	0.18	2005
2	Chinese Academy of Sciences	24	0.18	1998
3	China University of Mining & Technology	22	0.26	2013
4	Wuhan Institute of Rock & Soil Mechanics	16	0.12	2008
5	Central South University	15	0.01	2013
6	Northeastern University—China	14	0.05	2001
7	UDICE-French Research Universities	13	0.012	1997
8	Centre National de la Recherche Scientifique (CNRS)	12	0.10	1997
9	Dalian University of Technology	10	0.11	2005
10	Islamic Azad University	9	0.04	2014
	Hohai University	9	0.02	2015
	Shandong University	9	0.03	2013

**Table 3:** Top 10 journals by co-cited frequency.

NO.	Journal	Citation	Centrality	IF (2022)
1	INT J ROCK MECH MIN	262	0.04	6.849
2	ENG FRACT MECH	218	0.23	4.898
3	ROCK MECH ROCK ENG	200	0.00	6.518
4	INT J FRACTURE	187	0.05	2.635
5	INT J SOLIDS STRUCT	164	0.04	3.667
6	ENG GEOL	144	0.03	3.667
7	J GEOPHYS RES	107	0.06	/
8	J GEOPHYS RES-SOL EA	104	0.14	4.390
9	COMPUT GEOTECH	101	0.02	5.218
10	INT J NUMER METH ENG	92	0.02	3.021

**Table 4:** Top 5 highly cited references.

NO.	Title	Authors	Citation
1	Experimental Study on the Growth, Coalescence and Wrapping Behaviors of 3D Cross-Embedded Flaws Under Uniaxial Compression	Zhou, X.P. et al. [24]	31
2	Crack Propagation and Coalescence of Brittle Rock-like Specimens with Pre-existing Cracks in Compression	Cao, P. et al. [25]	17
3	An Experimental Study of Crack Coalescence Behaviour in Rock-Like Materials Containing Multiple Flaws Under Uniaxial Compression	Zhou, X.P. et al. [26]	16
4	Frictional crack initiation and propagation analysis using the numerical manifold method	Wu, Z.J. et al. [27]	15
5	Numerical simulation of propagation and coalescence of flaws in rock materials under compressive loads using the extended non-ordinary state-based peridynamics	Wang, Y.T. et al. [28]	15

## Data Availability

Not applicable.
